# Identification of viral infections in the prostate and evaluation of their association with cancer

**DOI:** 10.1186/1471-2407-10-326

**Published:** 2010-06-24

**Authors:** Margarita L Martinez-Fierro, Robin J Leach, Lauro S Gomez-Guerra, Raquel Garza-Guajardo, Teresa Johnson-Pais, Joke Beuten, Idelma B Morales-Rodriguez, Mario A Hernandez-Ordoñez, German Calderon-Cardenas, Rocio Ortiz-Lopez, Ana M Rivas-Estilla, Jesus Ancer-Rodriguez, Augusto Rojas-Martinez

**Affiliations:** 1 Department of Biochemistry and Molecular Medicine, University Hospital Universidad Autonoma de Nuevo Leon, (Av. F. I. Madero S/N, Col. Mitras Centro), Monterrey, (64460), Mexico; 2 Department of Urology, University Hospital Universidad Autonoma de Nuevo Leon, (Av. F. I. Madero S/N, Col. Mitras Centro), Monterrey, (64460), Mexico; 3 Department of Pathology, University Hospital Universidad Autonoma de Nuevo Leon, (Av. F. I. Madero S/N, Col. Mitras Centro), Monterrey, (64460), Mexico; 4 Department of Forensic Medicine, University Hospital Universidad Autonoma de Nuevo Leon, (Av. F. I. Madero S/N, Col. Mitras Centro), Monterrey, (64460), Mexico; 5 Department of Cellular and Structural Biology, University of Texas Health Science Center at San Antonio, (7703 Floyd Curl Drive), San Antonio, TX (78229-3900), USA; 6 Department of Urology, University of Texas Health Science Center at San Antonio, (7703 Floyd Curl Drive), San Antonio, TX (78229-3900), USA; 7 Department of Pediatrics, University of Texas Health Science Center at San Antonio, (7703 Floyd Curl Drive), San Antonio, TX (78229-3900), USA; 8 Unidad Academica de Medicina Humana y Ciencias de la Salud, Universidad Autonoma de Zacatecas, (Carretera Zacatecas-Guadalajara, Km 6. Ejido La Escondida), Zacatecas, (98160), Mexico; 9 Centro de Investigación y Desarrollo en Ciencias de la Salud, Universidad Autonoma de Nuevo Leon, (Calle Carlos Canseco S/N, Col. Mitras Centro), Monterrey, (64460), Mexico

## Abstract

**Background:**

Several viruses with known oncogenic potential infect prostate tissue, among these are the polyomaviruses BKV, JCV, and SV40; human papillomaviruses (HPVs), and human cytomegalovirus (HCMV) infections. Recently, the Xenotropic Murine Leukemia Virus-related gammaretrovirus (XMRV) was identified in prostate tissue with a high prevalence observed in prostate cancer (PC) patients homozygous for the glutamine variant of the RNASEL protein (462Q/Q). Association studies with the R462Q allele and non-XMRV viruses have not been reported. We assessed associations between prostate cancer, prostate viral infections, and the RNASEL 462Q allele in Mexican cancer patients and controls.

**Methods:**

130 subjects (55 prostate cancer cases and 75 controls) were enrolled in the study. DNA and RNA isolated from prostate tissues were screened for the presence of viral genomes. Genotyping of the RNASEL R462Q variant was performed by Taqman method.

**Results:**

R/R, R/Q, and Q/Q frequencies for R462Q were 0.62, 0.38, and 0.0 for PC cases and 0.69, 0.24, and 0.07 for controls, respectively. HPV sequences were detected in 11 (20.0%) cases and 4 (5.3%) controls. XMRV and HCMV infections were detected in one and six control samples, respectively. The risk of PC was significantly increased (Odds Ratio = 3.98; 95% CI: 1.17-13.56, p = 0.027) by infection of the prostatic tissue with HPV. BKV, JCV, and SV40 sequences were not detected in any of the tissue samples examined.

**Conclusions:**

We report a positive association between PC and HPV infection. The 462Q/Q RNASEL genotype was not represented in our PC cases; thus, its interaction with prostate viral infections and cancer could not be evaluated.

## Background

The contribution of immune and inflammatory responses to the development of cancer has been well recognized in different human tumors [1]. Viral infections may lead to chronic or recurrent inflammation of the prostate and initiate or promote carcinogenesis [2-6]. Infections of the prostate with polyomaviruses (BK, JC, and SV40), human papillomaviruses (HPVs), and members of the herpesvirus family (HHV-8, HCMV, Epstein Barr virus) have been previously described [4,7-11]. Viral products such as the large T antigen of polyomaviruses, or the E6 and E7 proteins of HPVs are able to induce cell transformation and interact with the signaling capacity of the interferon pathway in a synergistic manner [10].

The inflammatory etiology of prostate cancer (PC) is supported by the fact that the candidate gene for hereditary PC at the HPC1 locus is RNASEL, which is involved in antiviral and antiproliferative roles of interferons [12-15]. The R462Q variant of the RNASEL gene has been reported to occur in 13% of sporadic cases of PC [16]. The enzyme activity of this variant is reduced about two-thirds and this may impact cellular response against viral infection [17]. The RNASEL variant R462Q is suggested to increase susceptibility for PC and has been associated with an increase in prevalence of the Xenotropic Murine Leukemia Virus-related gammaretrovirus (XMRV) [7,9,11]. No study has reported relationships among the variant, other viral infections, and PC. In the present study the association between viral infection, prostate cancer and the RNASEL variant are assessed.

## Methods

### Patients and samples collection

The study was approved by the institutional review board of University Hospital of Universidad Autonoma de Nuevo Leon (ID number: B104-001/B107-001). All patients provided written informed consent prior to participation.

We enrolled one hundred thirty subjects who underwent transrectal biopsy (TRB) following confirmed clinical criteria [serum prostate specific antigen (PSA) value ≥4 ng/mL or abnormal digital rectal examination (DRE)], or transurethral resection (TURP) at our institution, between October 2006 and July 2007 for this population based case-control study. Criteria guidelines for TURP were: refractory urinary retention (at least failure in one attempt of catheter removal), recurrent urinary infection secondary to benign prostatic hyperplasia (BPH), recurrent macrohematuria secondary to BPH, bladder calculi secondary to prostatic enlargement, renal insufficiency secondary to BPH, and large or multiple diverticuli secondary to BPH. The case group included all subjects with histopathologic diagnosis of PC. The control group consisted of subjects who underwent a TRB or TURP, but had no pathological evidence of PC.

Venous blood samples for PSA determination and DNA extraction were drawn from each participating subject before their procedure. A questionnaire consisting of demographic data, risk factors, and urologic information was administered. PSA concentration was determined from serum samples by the IMMULITE 1000 PSA solid-phase chemiluminiscent immunometric assay. Sample collection was performed under local anesthesia and through the standard octant technique using an 18 gauge biopsy needle and a biopsy gun and eight cylinders were taken for histological diagnosis. Two additional cylinders from the right and left prostate lobes were taken for viral sequence identification. Fragments of 0.5 × 0.5 cm were taken from prostate tissue samples obtained by TURP for viral evaluation. All pathological examinations were performed by a single pathologist (RGG). Study subjects were not followed thereafter.

### Nucleic acid extraction

DNA from blood samples was isolated using a standard phenol/chloroform protocol, and DNA/RNA extraction of snap-frozen tissue samples was performed using the Trizol reagent (Invitrogen, Carlsbad, CA). Samples were homogenized and extracted according to the manufacturer's specifications. Quantity and quality of each extraction were determined using a spectrophotometer. PCR amplification of the GAPDH gene was performed to rule out the presence of PCR inhibitors.

### Genotyping

The evaluation of the R462Q variant (rs486907) of RNASEL was performed using the Taqman assay (Applied Biosystems: ABI, Foster City, CA), as reported by Urisman et al. [11]. In the first stage, 10 ng of DNA were amplified by allele-specific PCR. In a second step, allelic discrimination was made using the StepOne RT-PCR system with the StepOne v2.0 software (Applied Biosystems).

### Viral sequence detection

Before screening the prostate tissue for viral DNA, a validation stage for selected protocols for virus identification was performed to establish the copy detections limits. This was achieved through conventional PCR amplification, using serial dilutions of control plasmids for each virus consisting of 600,000 to 60 viral copies (Additional file 1: Table s1). The reaction products were electrophoresed through 3% agarose gels and visualized by ethidium bromide staining.

Viral sequences were identified in prostate tissue by nested PCR as previously described [4,8,11]. Briefly, 500 ng of DNA isolated from the prostate were subjected to PCR amplification designed to detect polyomaviruses and HPV [4,8,11]. For XMRV screening, 1 μg of RNA was reverse transcribed using the SSIII system (Invitrogen) and 2 μL of the cDNA reaction were used in the protocol described by Urisman et al. [11]. The HCMV detection was performed in two amplification rounds using sets of primers specific for the UL3 region, which produced amplicons of 420 and 188 bp, consecutively. The Additional file 1 provides information on primer sequences, regions, sizes of amplified products for each virus (Additional file 1: Table s1), and plasmids used as positive controls during the viral screening (Additional file 1: Table s2). For quality control purposes, pre-PCR, processes, PCR amplification, and post-PCR amplicon analysis were performed in separate areas, samples were run in duplicate, and negative controls were included in each experiment.

### Sequencing and viral Genotyping

PCR products from samples that were positive for viral sequences were purified by Wizard SV and PCR Clean-Up System (Promega, Madison WI). Direct sequencing was performed with the BigDye^® ^Terminator v3.1 Cycle Sequencing kit (Applied Biosystems) and analyzed using the Avant 3100 Genetic Analyzer. Sequences were compared with the reported reference sequences for each virus.

HPV DNA genotypes were distinguished using the Linear Array HPV Genotyping Test (Roche Diagnostics, Basel, Switzerland), according to the manufacturer's instructions and the test results were validated for two samples by sequencing.

### Statistical Methods

Cases and controls were compared using univariate methods. An independent t-test was performed for age and PSA comparison and the Mann-Whitney U test was used for comparisons of rank values of median values. Genotype distribution was checked for Hardy-Weinberg equilibrium by the exact test. A Chi square test was performed to test the relationship between Genotype-PC, Genotype-Infection and Infection-PC. Multivariate logistic regression was used with PC as the dependent variable. Two-sided p values < 0.05 were considered statistically significant.

## Results

### Clinical characteristics of the study sample

A total of 130 men (55 prostate cancer cases and 75 controls) were enrolled in the study. The majority (92.3%) of the subjects were from northwestern Mexico (states of Zacatecas, San Luis Potosi, Tamaulipas, Coahuila, and Nuevo Leon). For men with PC, 47.3% were older than 70 years of age while 24.0% of controls were older than 70 years. The mean age for cases was 71 years (range: 36-88) and the mean age for controls was 66 years (range: 50-88), p = 0.003. There was a statistical difference in PSA values between case and control groups (p < 0.001). The median PSA value was 18.30 ng/ml (range: 0.16-1062.00 ng/ml) for cases and 6.70 ng/ml (range: 0.20-25.99 ng/ml) for controls. Most men with PC had PSA values >10.0 ng/ml (Table 1).

**Table 1:** Comparison of study groups by PSA value and Gleason score

PSA value (ng/ml)	Cases	Controls	Total	Gleason score (frequency)
0.1 - 4.0	5	24	29	**6**(2), **9**(1), **10**(2)
4.1 - 8.0	4	27	31	**6**(3), **9**(1)
8.1 - 10.0	4	6	10	**6**(1), **8**(2), **9**(1),
>10.0	42	18	60	**6**(5), **7**(9), **8**(10), **9**(14), **10**(4)
Total	55	75	130	55

The most common pathological diagnosis in the control group was chronic prostatitis (84%), which was found in association with normal prostate tissue (41%), hyperplastic prostatic tissue (28%), and atrophic tissue (15%). Only 10 subjects (13%) had a histological diagnosis of normal prostatic tissue. As listed in Table 1, all PC cases have Gleason scores ≥6, with 35 (63.6%) having scores >7. PSA values ≤10 ng/ml were observed in 13/55 (23.6%) of men with Gleason scores ≥6. Five cases had PSA values ≤4.0 ng/ml, three of them with Gleason scores ≥9.

### R462Q Genotyping

All DNA/RNA samples demonstrated amplification of the GAPDH gene and were therefore used for subsequent analyses. Frequencies of the RNASEL R462Q genotypes R/R, R/Q, and Q/Q were 0.62, 0.38 and 0.0 for cases and 0.69, 0.24 and 0.07 for controls, respectively. Genotype frequencies for the control group did not depart from Hardy-Weinberg equilibrium by the exact test (p= 0.1), and due to the lack of representation of the Q/Q genotype in the PC cases, the association between the RNASEL R462Q variant and PC could not be determined. We also tested the association between PC or viral infection and R462Q genotype. For this association, frequencies of the R/Q and Q/Q genotypes were grouped and compared with the R/R frequencies. No associations with PC (p = 0.48) or viral infection (p = 0.34) were found in this subset analysis.

### Viral sequence detection

Specific PCR sequencing assays were standardized for each virus using positive controls (see Material and Methods). Detection limits of nested PCR protocols were established on 30 copies for the polyomaviruses and 60 copies for the HPV, XMRV, and CMV (data not shown).

The presence of BKV, JCV, and SV40 was not detected in any of the 130 prostatic tissues examined. Viral genomes for XMRV and HCMV were detected in one (1.33%) and six (8.0%) control tissues respectively, but not in the prostate tumors. The presence of HPV sequences was detected in 15 subjects (11.5%) of which 11 (73.0%) of them were cases and 4 (27.0%) controls. Genotype distribution of XMRV, HCMV and HPV positive samples is outlined in Table 2. Nine HPV positive subjects with PC were homozygous R/R for the RNASEL R462Q genotype and two were heterozygous R/Q for the RNASEL R462Q variant. HPV infection significantly increased the odds of PC by 4.4 times in our population (95% CI: 1.33 to 14.80, p = 0.021). Due to the difference in age in the study groups, adjustments for age were included in the multivariate logistic regression model. The odds ratio for HPV infection and PC was 3.98 (95% CI: 1.17 to 13.56, p = 0.027).

**Table 2:** Genotype distribution of samples positive for viral detection

	CASES			TOTAL	CONTROLS			TOTAL
				
Virus (+)/Genotype	R/R	R/Q	Q/Q		R/R	R/Q	Q/Q	
Py (BK, JC, SV40)	0	0	0	0	0	0	0	0
HPV	9	2	0	11	4	0	0	4
HCMV	0	0	0	0	3	2	1	6
XMRV	0	0	0	0	1	0	0	1
TOTAL	9	2	0	11	8	2	1	11

Differences between infection with these viruses and serum PSA levels were not found (p = 0.9). There were no associations found between RNASEL R462Q genotype and the presence of any viral infection (p = 0.4).

Given the limited size of the tissue specimens, HPV sub-typing was performed on only six samples (five cases and one control) as shown in Table 3. The samples were screened for ten different types of HPV; seven of them were oncogenic high risk HPVs (33, 45, 52, 58, 66, 68, and 83) and three were oncogenic low risk viruses (44, 81, CP6108). Three samples showed infection with multiple HPV types, while the remaining three had a single HPV type infection. Oncogenic high risk HPVs were found in four of the five cancers and in the control.

**Table 3:** Summary of HPV typing and characteristics of clinical specimens

Group	Age	Gleason Score	PSA value (ng/ml)	HPV type
Case	81	8 (4+4)	47.1	58, 52, 66
Case	77	9 (4+5)	6.69	81
Case	57	10 (5+5)	26.2	45, CP6108
Case	79	10 (5+5)	0.16	68, 33
Case	75	9 (5+4)	100	44
Control	72	-	12.1	83

Some limitations of the study include the small sample size which may introduce bias into the statistical analyses performed. However, in most of the cases we used a low quantity of prostate tissue (two TRB cylinders) for the virus screening and it could to enhance the differences in virus detection between studies. We included 18 control subjects with serum PSA values ≥10 ng/ml, despite the high probability (≥50%) of detecting cancer in individuals who have PSA values ≥10 ng/ml [18]. Since most of these subjects did not have PC diagnosis in any of two consecutive TRB procedures, they were not excluded from the study. Their high PSA values may be related to a different pathology, such as prostatitis, hyperplasia, or atrophy, which may impact the PSA levels [19,20]. Moreover, exclusion of these subjects from the OR calculations confirms the association between HPV infection and PC (OR = 4.8, 95% CI: 1.25-18.37, p = 0.03).

## Discussion

Genetic factors, such as the R462Q variant of RNASEL, may play a role in the etiology of PC. In addition, viral infections of the prostate have also been suggested as important virally-related risk factors for PC, particularly, infections with HCMV, JCV and BKV, high risk HPV, and more recently, XMRV [4,5]. However, studies designed to replicate the association of either the R462Q variant or viral infections with PC have yielded conflicting results worldwide. This current study analyzed associations between viral infections, the R462Q variant of RNASEL, and PC. Our results showed a low frequency of Q/Q homozygous subjects (6.7% in controls and 0.0% in cases) compared with those found in previous studies (5.7-13.3%) [21] and associations with this genotype could not be demonstrated. This low genotype frequency may be explained by racial/ethnic variations.

Detection of viral genome sequences in prostatic tissue was restricted to HCMV (4.6%), XMRV (0.8%), and HPV (11.5%). The only XMRV positive sample was from a control subject with the RNASEL R462Q R/R genotype (64 years old, PSA value of 0.64 ng/ml, and normal prostate tissue by histology). The relationship between viral infection and the R462Q Q/Q genotype has been reported for the XMRV only, and thus no comparison had been made for other prostate viral infections.

A comparison on the prevalences of HPV, HCMV, polyomaviruses, and HPV in prostatic tissue (including ours) is shown in Table 4. HPV DNA sequences were found in 11/55 cases (20.0%) and 4/75 (5.3%) controls. This infection increases the risk of PC by 3.98 times (p = 0.027). These results are consistent with the data reported by Leiros et al. [22], who demonstrated a significant association between the presence of HPV sequences and prostate carcinomas in Argentina (41.5% prevalence in PC cases). Zambrano et al. [4] detected a high HPV prevalence in prostate tissue in six of 12 PC patients using PC tissue and urine specimens. Conversely two independent studies separately reported by Bergh and Sfanos in Sweden and the United States did not find HPV sequences in any of the 352 and 200 prostatic tissue samples screened, respectively [23,24] (Table 4).

**Table 4:** Comparative summary and main findings of prostate viral infection reports

Country/Study Description	Pathogen	Samples (+)	Total	Findings	Reference
Sweden/201 prostate tissue samples from patients with BPH that later progressed to PC and 201 matched controls that did not.	Epstein-Barr Virus. JCV. BKV, HPVs, CMV. Adenovirus, HSV1 and 2 *C.albicans*.	31 10 0 0 2	43 (352)	There were no differences in the prevalence of adenovirus, herpesvirus, papillomavirus, polyoma virus and *C. albicans *DNA.	Bergh J. British Journal of Cancer 2007.
Baltimore, USA/30 PC subjects for bacterial DNA and 200 PC cases for viral-parasite DNA detection.	BKV. *Chlamydia T.* CMV. Epstein-Barr Virus. HPVs, XMRV.	1 1 1 16 0	18 (200)	Most prostatectomies (87%) contain bacterial DNA from one or more species. The majority of individual tissue core samples were negative, suggesting regional heterogeneity in the presence of bacteria and a lack of a generalized or ubiquitous prostatic flora.	Sfanos KS. The Prostate 2008.
Greece/42 samples of prostatic malignancies.	BKV. JCV. HPVs..	8 0 2	10 (42)	BKV was frequently detected and could play a relevant role in the development and progression of human PC, whereas HPV does not seem to be implicated in this type of human neoplasia.	Balis V. Int J Biol Markers. 2007.
LongBeach, USA/ 12 PC Fresh frozen tissues and 20 paraffin-embedded archival samples.	HPVs. JCV. BKV.	0 (20), 6 (12) 10 (20),6 (12) 0 (20), 3 (12)	10 (20) And 7(12)	The prostate is a complex habitat where mixed infections with oncogenic DNA viruses frequently occur and opens the discussion to the potential role of these viruses in the PC.	Zambrano A, Prostate 2002.
Monterrey, Mexico/55 Cases of PC and 75 Controls.	JCV, BKV, SV40. HPVs. HCMV. XMRV.	0 15 6 1	22 (130)	HPV frequency was significantly higher in PC cases than in controls; this finding suggests their participation in PC develpment or tumor progression.	This work.

Discrepancies may be explained by technical differences in the viral detection methods or differences in sexual behavior in the study populations. HPV prevalences in healthy men demonstrate large variations worldwide, ranging between 3% and 45% [25-27]. There are no data available about prostate HPV infection in Mexico; however, our results are in accordance with previous reports of the high HPV prevalence (43-46%) in exfoliated cells from the urethra-coronal sulcus of sexually active subjects and healthy military men in Mexico, respectively [27].

We found different types of oncogenic high risk HPV without the presence of a predominant HPV type, suggesting that the HPV association with PC is not due to specific type of HPV, as implied in previous reports, particularly since HPV16 or HPV18 were not detected in this study [4,28]. The remarkable incidence of multiple HPV infections reported here, suggest a relationship with PC that deserves further analyses.

Most of the tissue samples used were TRB specimens. It has been suggested that viral contamination of prostate tissue by HPV and polyomaviruses (detectable in urethral or anal tissues), could occur from neighboring tissues during the sampling procedure; but differences in virus prevalence between case and control groups minimizes the contamination possibility.

It is well known that the first line of defense against viral infection is the interferon response, as E6 and E7 HPV proteins can inhibit the signaling capacity of interferon pathway [10]. This may be associated with disease aggressiveness. Our results indicated no association between HPV infection and tumor aggressiveness represented by Gleason score.

In this study, polyomavirus genomes were not detected in any of the 130 evaluated tissue samples; however, six HCMV positive samples were detected in the control group. Absence of HCMV, polyomaviruses, and XMRV in tumor prostate tissue suggests that they are not associated with PC in Mexican population, though positive control samples (with the exception of the XMRV's positive sample) demonstrated chronic prostatitis that may evolve to malignancy, as suggested by some authors [2-6].

Our results for the XMRV virus in a Mexican sample differ from those reported by Urisman et al. [11] who described 8 out of 20 XMRV positive samples in Q/Q homozygous subjects versus 1 out of 52 positive samples in R/R subjects. XMRV prevalence in sporadic PC cases has been notoriously lower than reported by Urisman. Fischer et al. [9] identified two XMRV positive samples in 157 prostatic tissue samples and none in eleven Q/Q homozygous tissue samples included in the study performed in German subjects. In an additional report from this country, Honh et al. [29] could not demonstrate the presence of the XMRV in 589 prostate tumor samples using real-time PCR. D'arcy et al. [7] reported no evidence of XMRV in Q/Q Irish PC patients. A recent report by Schlaberg et al. [30] describes no association between XMRV infection and RNASEL R462Q genotype; but in contrast, found association between retroviral infection and PC. Mutations in the RNASEL locus other than the R462Q variant may affect either the PC risk or the susceptibility to viral infections of the prostate, but they were not aimed in this study.

## Conclusions

We found association between PC and prostate infection by HPV. The 462Q/Q RNASEL genotype was not represented in PC cases, therefore the interactions among prostate viral infections, PC, and the 462Q allele could not be proven.

## Competing interests

The authors declare that they have no competing interests.

## Authors' contributions

Study Design: ARM, MLMF, RJL, and LSGG. Data acquisition: MLMF, LSGG, RGG, IBMR, MAHO, GCC, and JAR. Data analysis and interpretation: MLMF, RJL, LSG, RGG, TJP, JB, ROL, AMRE, and ARM. Statistical Analysis: MLMF, and JB. Manuscript preparation: MLMF, RJL, JB, ROL, AMRE, and ARM. Manuscript review: MLMF, RJL, JB, ROL, TJP, and ARM.

All authors read and approved the final manuscript.

## Pre-publication history

The pre-publication history for this paper can be accessed here:

http://www.biomedcentral.com/1471-2407/10/326/prepub

## Supplementary Material

Additional file 1 **Additional file 1 includes primer sequences and positive control information used for the virus screening experiments **(Additional file 1: Table s1 and s2, respectively). Click here for file
