# Supplementary material for: Identification of viral infections in the prostate and evaluation of their association with cancer
Source: BMC Cancer. 2010 Jun 24;10:326. doi: 10.1186/1471-2407-10-326 (PMC2912861; doi:10.1186/1471-2407-10-326)
Supplement: Additional file 1 — includes primer sequences and positive control information used for the virus screening experiments (Additional file 1: Table s1 and s2, respectively). [file 1471-2407-10-326-S1.PDF]

Additional file 1.

1. Table s1. Primers information.

Name	Gene/Region	Sequence	Size of PCR product
GAPDH	GAPDH	5'-GAAGGTGAAGGTCGGAGTC-3'<br>5'-GAAGATGGTGATGGGATTTC-3'	225 bp
GAG O	GAG	5'-CGCGTCTGATTTGTTTTGTT-3'<br>5'-AGAGGGTAAGGGCAGGGTAA-3'	413 bp
GAG I		5'-TCTCGAGATCATGGGACAGA-3'<br>5'-CCGCCTCTTCTTCATTGTTC-3'	290 bp
HCMV1	UL3	5'-CAAGCGGCCTCTGATAACCAAGC-3'<br>5'-CTCTTCCTCTGGGGCAACTTCCTC-3'	420 bp
HCMV 2		5'-CCGATCCTCTGAGAGTCTGCTCTC-3'<br>5'-CAGCCACAATTACTGAGGACAGA-3'	188 bp
MY09<br>MY11	L1	5'-CGTCCMARRGGAWACTGATC-3'<br>5'-GCM AGGGWCATAAYAATGG-3'	450 bp
GP5+<br>GP6+		5'-TTTGTTACTCTGGTAGATACTAC-3'<br>5'-GAAAAATAAACTGTAAATCATATTC-3'	150 bp
PM1F<br>PM1R	T-Antigen	5'-TCYTCTGGNNTAAARTCATGCT-3'<br>5'-AAWTAGRTKCCAACCTATGGAA-3'	550 bp
PM2R		5'- GGTAGAATACCAAATGACTTTCC-3'	
BKF		5'-GAATGCTTTCTTCTATAGTATGG-3'	353 bp
JCF		5'-ATATTATGACCMCCAAAACCATG-3'	189 bp
SVF		5'-ATAATTTTTTTGTATAGTAGTGCA-3'	135 bp

2. Table s2. Positive control information.

Virus	Control (+)	Type/var	ATCC No.
**BKV**	pBKV33-1	BK Virus Gadner	45024
**JCV**	pJCV	Mad1	45027
**SV40**	pBRSV	SV40 776	45019
**HCMV**	pHCMV†	Towne	
**XMRV**	pXMRVm†	-	
**HPVs**	pBRg HPVs‡	11,16,18,33,53.	

† Plasmid control generated in this study: pHCMV from HCMV genome (ATCC number: VR-977), and pXMRVm from DNA of 22Rv1 cellular line respectively. ‡ HPV genomes donated by the Unit of Molecular Diagnosis, UANL.
